# Assessment of shoulder functional movements through inertial measurement units for tele-rehabilitation: a quaternion-based approach

**DOI:** 10.3389/fdgth.2025.1576031

**Published:** 2025-09-01

**Authors:** Matteo Iurato, Paolo Dondero, Mirko Job, Ronny Stanzani, Gaia Leuzzi, Igor Ingegnosi, Marco Testa

**Affiliations:** ^1^ REHELab, Dipartimento di Neuroscienze, Riabilitazione, Oftalmologia, Genetica e Scienze Materno-Infantili, Università degli studi di Genova, Genova, Italy; ^2^ Swhard s.r.l., Genova, Italy; ^3^ Department of Physical Education and Rehabilitation, Experimental Anatomy Research Group (EXAN), Vrije Universiteit of Brussel (VUB), Brussels, Belgium

**Keywords:** IMU, movement assessment, quaternions, rehabilitation exercises, shoulder

## Abstract

Telerehabilitation improves accessibility and accelerates recovery: in this context, Inertial Measurement Units (IMUs) are promising wearable sensors for remote movement data collection, which allows to evaluate how closely exercise repetitions align with a prescribed trajectory. Current data processing methods for this purpose include data-driven approaches, requiring exercise-specific training through large amount of data, or distance-based methods with unbounded output, not easy to interpret. This study proposes a novel algorithm which combines the versatility of a bounded output score with numerical stability of quaternions. Data from an IMU-based device were acquired during the execution of human functional shoulder movements by both a young and elderly group of participants. Outputs from the application of the proposed methodology on collected data from same or different movements were statistically compared, revealing ability of discriminating repetitions of the same or of different movements (p<0.01, *rrb* effect size = 0.97, contrast ratio 1.7). The proposed algorithm was also confronted with the traditional approaches by statistically comparing outputs from comparison matrices rescaled in equal range of values, and results indicated mild differences in performance (*rrb* effect size < 0.5). Future works may involve integrating this approach into a functioning telerehabilitation system and obtaining feedback on the usability from real users.

## 1 Introduction

The shoulder joint is one of the most complex structures in the human body (1) and is one of the most frequently affected by musculoskeletal disorders (MSDs) (2). MSDs significantly impact the quality of life of those affected (3) and represent an economic and logistic burden for the healthcare system (2, 4–6). This is mainly due to the prolonged rehabilitation treatment required for full recovery (4). Rehabilitation is indeed often performed in clinical settings, but the possibility of pursuing the treatment at home would allow patients to save time and money, and healthcare structures to simplify procedures and contain costs (4–7). Therefore, the interest in developing home-based rehabilitation solutions has grown over time (4).

Nonetheless, home-based rehabilitation is often jeopardized by the patient’s low adherence to the prescribed treatment owing to the absence of expert supervision (8). Ensuring the patient’s adherence to a prescribed movement and its correct execution represent key elements that can speed up the rehabilitation process (9–13).

To this extent, data from motion capture sensors need to be processed to provide both the patient and clinician with intuitive feedback about the appropriateness of the executed movement.

Among motion capture technologies, the most suitable for telerehabilitation are Inertial Measurement Units (IMUs) (14–19): these types of sensors can indeed be easily worn and attached to a person, measuring joint angles and accelerations without the need for any additional devices or supervision (15).

A successful algorithm for processing data from IMUs to provide feedback on correct movement execution in a telerehabilitation system should therefore account for two factors: numerical stability and patient engagement.

Numerical stability is highly influenced by the representation used to describe three-dimensional kinematics. Kinematic description of movements of the shoulder joint involves not only assessing basic planar movements (10, 20, 21), but also functional movements performed in daily life that might be impeded by impairments (10, 22–24). In this context, the most numerically stable representation is provided by quaternions, due to the singularity phenomena inherent to other three degrees of freedom representations (25, 26). Moreover, quaternions are the output of most sensor fusion algorithms that extract orientation from raw IMU data (27), which makes them a convenient representation to be used for assessing correct execution of movements in telerehabilitation systems.

Patient engagement is usually obtained by processing data to obtain an indicator of movement conformity to a template. Some methods provide discrete indicators (e.g., correct/incorrect) (28–30), which, however, do not provide a continuous scale of correctness, impeding the patient to understand the degree of error (31). Other methods involve data-driven approaches (31): this is a very powerful methodology, which, however requires large dataset that are exercise-specific to be trained, and therefore does not allow flexibility in changing the movement being assessed without training on new datasets (31, 32).

Another possibility involves using distance-based algorithms to compare trajecotries (31, 33), which is a very operationally simple technique, requiring no a priori knowledge nor training phase (31). Nonetheless, most of the studies exploring this approach use distance-based algorithms with unbounded outputs, which makes the output not easy to interpret and less intutive. The potential of using distance-based algorithms providing an output in a bounded range seems underexplored.

In this study, we present an approach for the evaluation of shoulder functional movements that is quaternion-based for numerical stability and relies on a new bounded algorithm for the trajectory similarity measure. Next, we present results from the verification of the approach using data collected through an IMU-based device developed by our research group (34) and comparing its performance with that of existing algorithms.

The contributions of the study are the following:

• To define an assessment methodology in quaternion space with the aim of improving numerical stability with respect to other representations of kinematics
• To provide a novel easy-to-use (no training required), flexible (not exercise-dependent) and easy to understand (bounded score) algorithm to compare quaternion trajectories and compare its performance with the one of existing algorithms
• To verify the joint approach involving quaternion analysis and trajectory comparison through bounded output distance-based algorithms using IMU data related to shoulder functional movements, with the aim of enhancing the potential of IMUs in wearable devices for remote monitoring by promoting their use in a functional approach to telerehabilitation

## 2 Related works

The analysis of human movement plays a crucial role in many different fields (35). However, traditional movement analysis techniques such as photogrammetry, optoelectronic analysis and video analysis, require complex and expensive instrumentation (36). Furthermore, these systems are characterized by low portability, which limits their applications outside the laboratory environment (14). As a first step towards the applicability of motion capture systems for remote monitoring of movement, some studies explored assessing the quality of movement combining Virtual Reality systems and kinematic models (37, 38): however, also these systems require the use of cameras with markers or controllers and head-mounted displays.

In recent years, significant progress has been made in the development of Inertial Measurement Units (IMUs). As a result, researchers have suggested the use of these devices to overcome the limitations of conventional movement analysis systems, especially when data needs to be collected in ecological settings (14, 15). IMUs are widely used in the most recent studies for movement analysis, since this type of sensors offers the possibility to be easily worn and attached to the person, measuring joint angles and accelerations without the need for any other additional devices (14–19).

Thanks to the potential of recent motion capture technologies to allow for the development of telerehabilitation systems, literature about data processing for remotely assessing conformity of movement has grown over time.

One first aspect to consider when assessing the conformity of movement is how to represent the kinematics. Because recent techniques for shoulder rehabilitation not only involve simple planar movements, but also complex three-dimensional functional movements (11, 12, 39), for these complex movements the evaluation of the Range of Motion (RoM) on a plane (20, 24, 40, 41) is not sufficient, and an analysis of the overall three-dimensional kinematics is needed. However, representation of rotations through three degrees of freedom approaches (e.g., Euler or Cardan angles) is very prone to errors due to phenomena such as singularities and gimbal lock (25, 26). Therefore, even if it is less intuitive, quaternion representation is recommended to enhance numerical stability. Moreover, quaternions are the output of most sensor fusion algorithms that extract orientation from raw IMU data (27).

The second aspect to consider for movement conformity assessment is the algorithm used to compare trajectories. A review of the available computational approaches for evaluating rehabilitation exercises has been provided by Liao et al. (31). They have classified assessment techniques into three categories: discrete movement score, rule-based, and template-based approaches (31).

Discrete movement score approaches evaluate movement based on discrete classes (e.g., correct/incorrect): despite being a first good indicator of correctness, this might be frustrating for the patient owing to the unavailability of finer information about the level of correctness, which could instead be provided through a continuous assessment scale (31).

Rule-based approaches rely on the a priori definition of a set of rules for exercise execution: despite being a well-performing approach in many contexts, it lacks generality and therefore forces the evaluation system to be exercise-specific, leaving little flexibility to the clinician who has to re-define the rules if the therapeutic approach changes (31).

Finally, in the template-based approach, a series of exercise repetitions is compared with a template (reference) movement, and a quantitative indicator of coherence with respect to the reference is provided. This approach both allows personalized patient-based therapy (by defining a patient-specific template) and fosters patient enhancement by providing engaging indicators. The comparison of repetitions with the reference template is very often performed using probabilistic approaches, such as Hidden Markov Models (42–45) or deep learning algorithms (e.g., Graph Convolutional Networks) (46). This approach is very powerful, although it requires the collection of sufficiently large datasets to be trained (32), partially leading to the loss of flexibility in defining patient-specific therapy.

Another approach for comparing repetitions with the reference template is using distance metrics, such as Euclidean or Mahalanobis distance (42, 43, 47, 48). These techniques are very easy to implement if signals have the same length and are uniformly sampled (i.e., all movement repetitions have the same duration and are performed at the same speed) (31, 48): in this case, the use of Euclidean distance is defined as lock-step Euclidean distance (point-to-point distance measure) (49). However, if a constraint on the speed is not imposed, more elaborated trajectory comparison methods need to be considered.

A comparative analysis of algorithms for measuring trajectory similarity was provided by Tao et al. (49). Specifically, they considered five methods: dynamic time warping (DTW) (50–52), Fréchet distance (FD) (53), discrete Fréchet distance (DFD) (54), edit distance on real sequence (EDR) (55) and longest common subsequence (LCSS) (56). All these methods allow the comparison of trajectories of different lengths and non-uniformly sampled, using two main different approaches: DTW, FD and DFD are a variation of the basic lock-step Euclidean distance aimed at considering different lengths and non-uniform sampling; therefore, these methods provide as an output an unbounded measure of similarity. On the other hand, EDR and LCSS define a threshold below which points in the compared trajectories are considered similar; consequently, the output of these algorithms is a bounded similarity measure, related to the number of points classified as similar.

Unbounded algorithms, especially DTW, are often used when assessing rehabilitation exercises because of their proven reliability (31, 33). Nonetheless, to make the unbounded output intuitive for the clinician and engaging for the patient, it is necessary to convert it into a conformity score, which is usually done by: relying on information about data estabilished a priori or inferred from large datasets (e.g., bound limits) (43, 57–59), familiarization procedures for the clinician and the patient (60), or output discretization (61, 62). This may limit the potential versatility of the template-based approach.

Bounded algorithms rely on determining the similarity of points in trajectories based on an acceptance threshold; therefore, they can directly provide a conformity score as an output without a priori knowledge of the movement. However, their potential in telerehabilitation applications remains underexplored.

These premises led to the development of a quaternion-based approach with trajectory comparison performed through a novel bounded algorithm aimed at assessing repetitions of functional complex movements of the shoulder joint, which is presented in this study.

## 3 Materials and methods

### 3.1 Mathematical description of the proposed methodology

In this section, we provide detailed mathematical description of the proposed methodology for assessing coherence between repetitions of functional movements using analysis in quaternion space and bounded distance-based algorithms for trajectory comparison.

Considering the acquisition of a quaternion over time, it is first necessary to calculate the spatial trajectories resulting from the combination of quaternion components. In the following, a quaternion q is denoted as in Equation 1:

(1) $$q=q_{w}+iq_{x}+jq_{y}+kq_{z}$$

where w, x, y, z are the four components of the quaternion and i,j,k denote the imaginary parts (63).

To analyze the trajectories in the quaternion space, we evaluated the bi-dimensional correlations among the four components of a quaternion. To this extent, by coupling the components, we analyzed the following bi-dimensional trajectories:

(2) $$y_{\left(x\right)},z_{\left(x\right)},z_{\left(y\right)},w_{\left(x\right)},w_{\left(y\right)},w_{\left(z\right)}$$

The six trajectories listed in Equation 2 represent the six combinations of the four quaternion components in couples (all possible couple combinations of four elements with no repetition).

After obtaining the spatial trajectories in quaternion space, it is necessary to compare the various repetition of a movement with a template trajectory. To this extent, we applied trajectory comparison algorithms (49) to each of the quaternion components trajectories listed in Equation 2, comparing the trajectory of the repetition with that of the template. Next, to provide a synthetic indicator of coherence between the two movements, we averaged the outputs from the six comparisons.

For the comparison of the quaternion components trajectories, we considered a novel algorithm called Nearest Neighbor Score (NNS) and compared its performance with the trajectory comparison methods described in (49) (DTW, FD, DFD, EDR, and LCSS).

The implementation of the new NNS algorithm is described hereafter. We implemented the algorithm in Python 3.11, with Numpy 1.26 and Pandas 2.1 for calculations, through a routine suitable for every platform and operating system. See data availability statement for reference to the code repository. However, hereafter, we explain the concept behind the implementation and we present the pseudo-code so that the steps necessary to calculate the conformity score can be replicated in any other programming language.

The trajectories compared through the algorithm are in the following denoted as: $b_{\left(a\right)}$ for the template movement with length *n*, $b_{\left(a^{\prime }\right)}^{\prime }$ for the repetition movement with length *m*. For each point in the *template* trajectory, the algorithm:

1. finds the two nearest points in the *repetition* trajectory;
2. calculates the interpolating line between these two points;
3. finds the b-value on the interpolating line at the same a as the *template* point;
4. calculates the distance between the b-value of the point on the interpolating line and the b-value of the *template* point;
5. increments a *conformity score*, expressed as a percentage of the number of samples *n*, if the distance calculated in the previous step exceeds one of the following thresholds:
  • $b_{t}$, defined as the 20% of the absolute range of the *target* curve (value chosen by considering a ± 10% tolerance around the considered point)
  • $t_{\mathrm{acc}}$, which was set based on the accuracy of the measurement instruments used.

**Figure 1:**
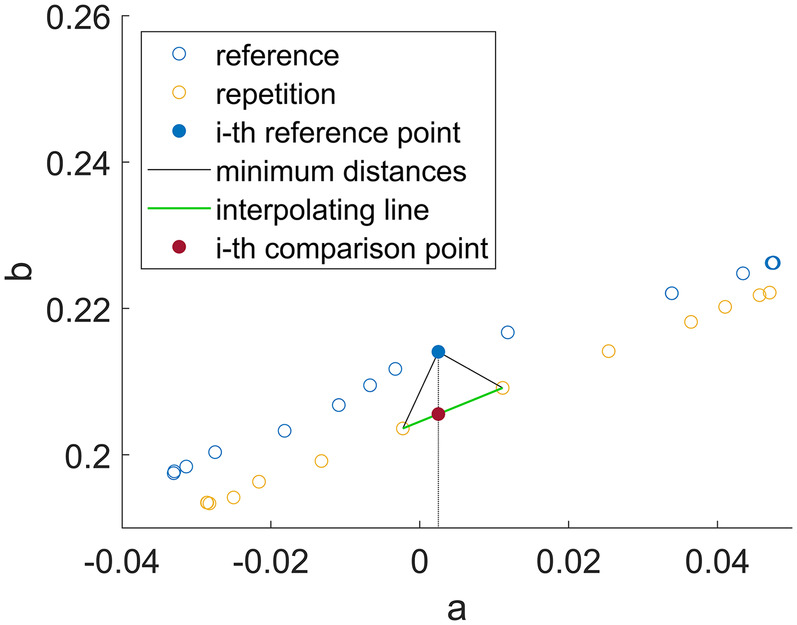
Schematic illustration of the steps of the NNS algorithm for one point in the template trajectory.

Figure 1 illustrates the described process, and Algorithm 1 provides the pseudo-code implementation.

**Algorithm 1:** Pseudo-code implementation of the NNS algorithm.

Define threshold $b_{t}$ Define threshold $t_{\mathrm{acc}}$ Initialize score s=0 **for** i=1 **to** n **do** **for** j=1 **to** m **do** Calculate Euclidean Distance $d_{ij}=\sqrt{(a_{\;j}^{\prime }-a_{i}{)}^{2}+(b_{\;j}^{\prime }-b_{i}{)}^{2}}$ **end** Find the two smallest *d*_*ij*_ and the respective points $b_{n1}^{\prime }=b_{\left(a_{n1}^{\prime }\right)}^{\prime }$, $b_{n2}^{\prime }=b_{\left(a_{n2}^{\prime }\right)}^{\prime }$ Interpolate linearly $h_{i}=\frac{a_{i}-a_{n2}^{\prime }}{a_{n1}^{\prime }-a_{n2}^{\prime }}b_{n1}^{\prime }-\frac{a_{i}-a_{n1}^{\prime }}{a_{n1}^{\prime }-a_{n2}^{\prime }}b_{n2}^{\prime }$ **if** $|h_{\left(a_{i}\right)}-b_{\left(a_{i}\right)}|<t_{\mathrm{acc}}$ **then** Increment score s=s+1 **end** **else if** $|h_{\left(a_{i}\right)}-b_{\left(a_{i}\right)}|<b_{t}$ **then** Increment score s=s+1 **end** **end** Express score as a percentage of the number of samples $s^{\%}=\frac{s}{n}\cdot 100$

In the final step of the algorithm, the score is expressed as a percentage of points classified as similar (based on the thresholds) over the total number of points. This implies that an output below 50% means that there were less points classified as similar than points classified as different. On the opposite, a score greater than 50% means more similar point than different points, and the closer the score gets to 100%, the higher is the number of similar points.

### 3.2 Data collection

This section illustrates the methodologies for the data collection performed in order to verify the proposed approach.

Orientation data were acquired through an IMU-based wearable device, called Sentry, developed by *Swhard s.r.l.* in collaboration with the *REHELab* (University of Genoa, Italy). The device incorporates two BNO080 sensors, produced by a collaboration between Hillcrest Labs and Bosch Sensortec GmbH (64), each one integrating a 14-bit tri-axial accelerometer, a 16-bit 3-axis gyroscope and a geomagnetic tri-axial sensor, accompanied by a 32-bit ARM Cortex®-M0 microcontroller running a proprietary software (34). The accuracy, reliability, and repeatability of the device measurements were previously demonstrated through tests involving movements on a robotic arm (34). The output from the sensors are two unitary quaternions over time, representing their orientation expressed in the earth Reference Frame. Data are transmitted via Bluetooth at a sampling frequency of 25 Hz to a personal computer. On the computer, an acquisition and storage software platform (customly developed Microsoft Universal Windows Platform) saves the data as plain text files.

A first set of tests was carried out with a robotic arm, using the same setup as described in (34), to test the algorithm under controlled conditions, simulating basic shoulder movements. One sensor was fixed as a reference on the table, and the other was mounted on the robotic arm. In this scenario, three movements were considered as the *template*: $30^{\circ }$ abduction, $50^{\circ }$ flexion, and $10^{\circ }$ external rotation, performed at a constant speed of $25^{\circ }$/s. Ten *repetitions* for each of these three movements were executed at varying speeds of $10^{\circ }$/s (*slow*), $25^{\circ }$/s (*normal*), and $50^{\circ }$/s (*fast*) and were compared with the respective *template*. The movement amplitude was chosen based on a compromise between the amplitudes commonly achieved in everyday movements and the possibilities offered by the robot’s workspace. The speed values were selected based on the technical capabilities of the robot. Examples of the angular and quaternion trajectories are provided in Supplementary Material.

**Figure 2:**
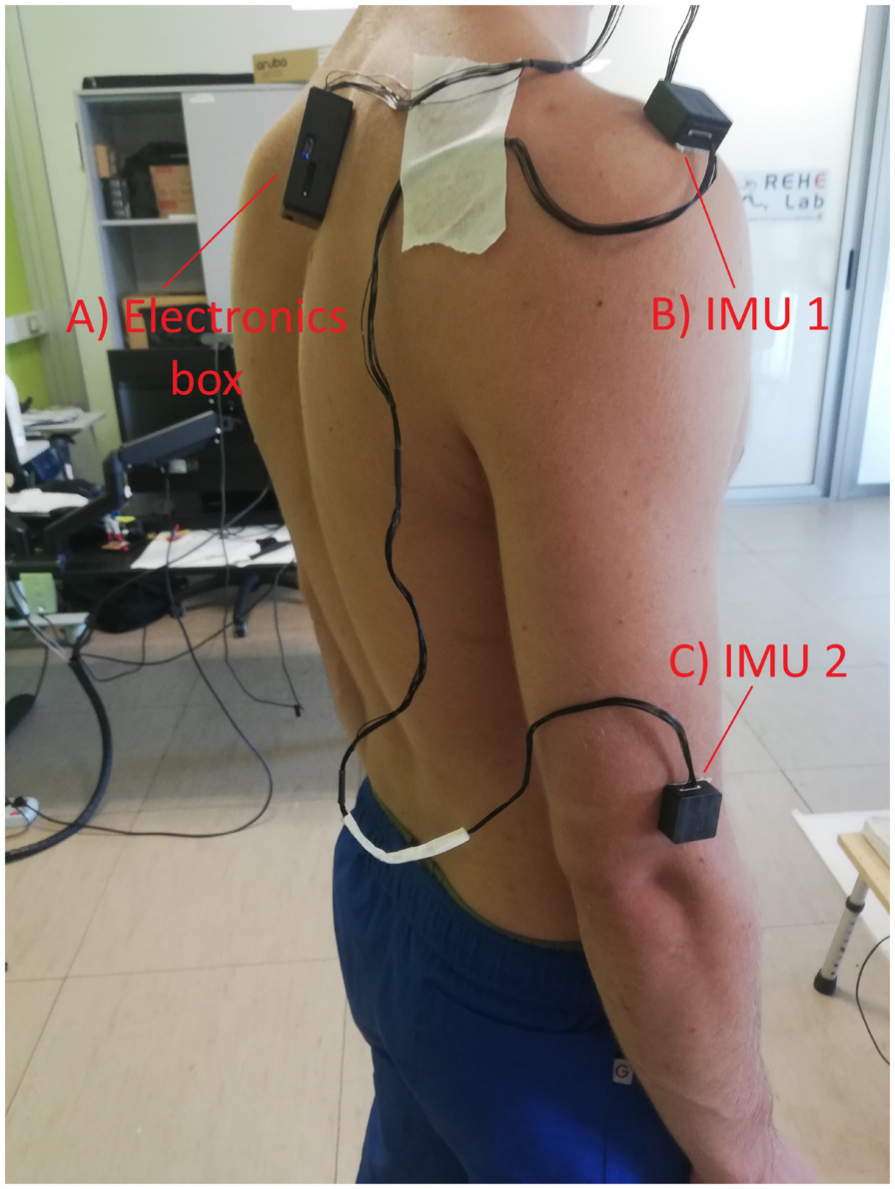
On-person sensors positioning.

After testing on the robotic arm, the approach was applied to shoulder movement data replicating activities of daily living (ADL) acquired from human participants. We included right-handed adults with no history of shoulder surgery or lesions from two different age groups (one ranging between 20 and 30 years old—in the following denoted as the young group, and the second between 60 and 85 years old—in the following denoted as the elderly group), as approved by the Ethical Committee for University Research of the University of Genoa (CERA n. 2023/78). Six ADL-related movements—upper care, medium care, lower care, driving a car, frontal reaching, and upper reaching—were retraced based on (65) (Table 1). Exercises were defined as either non-standardized (NST) with unrestricted shoulder RoM or standardized (ST) with RoM constrained by verbal instructions (see Table 1). See Supplementary Material for examples of the angular and quaternion trajectories. The participants freely performed ten repetitions of each exercise in a randomized order. The IMUs were placed on the Acromion and 4 cm above the Epicondyle (Figure 2). For each movement, a randomly selected repetition was used as the *template* to which compare the other nine repetitions.

**Table 1:** Functional movements considered in the experiment, divided in standardized (ST) and non-standardized (NST). All movements start in anatomical position (rest position). For each movement, the associated ADL is reported.

Name	NST movement	ST movement	Associated ADL
Upper care	With the hand: reach forehead, reach nape, go back to forehead, return to rest	With the hand: reach forehead keeping the elbow in contact with trunk, reach nape while opening the elbow outside, go back to forehead while closing the elbow inside, return to rest keeping the elbow in contact with trunk	Washing head
Medium care	With the hand: reach contralateral shoulder, go behind neck, reach wrist, go back to shoulder, go behind neck, return to rest	Same as NST, but keeping the elbow in contact with trunk whenever possible	Washing arm
Lower care	With the hand: reach contralateral gluteus	Same as NST, but keeping the hand in contact with the body	Picking an object from the opposite pocket
Frontal reaching	Bring the hand in front of the sternum at shoulder height, bring the hand to the mouth, return to rest	Same as NST, but keeping the elbow in contact with trunk whenever possible	Picking food in front of you and feeding yourself
Upper reaching	Bring the elbow in front of the sternum at eyes height with the arm extended, bring the hand to the mouth, return to rest	Same as NST, but keeping the elbow in contact with trunk whenever possible	Picking food upon a shelf and feeding yourself
Driving		Bring both hands at shoulder height with the arm extended, rotate $90^{\circ }$ right, return in the previous position, rotate $90^{\circ }$ left, return in previous position	Driving

### 3.3 Data processing and statistical analysis

**Figure 3:**
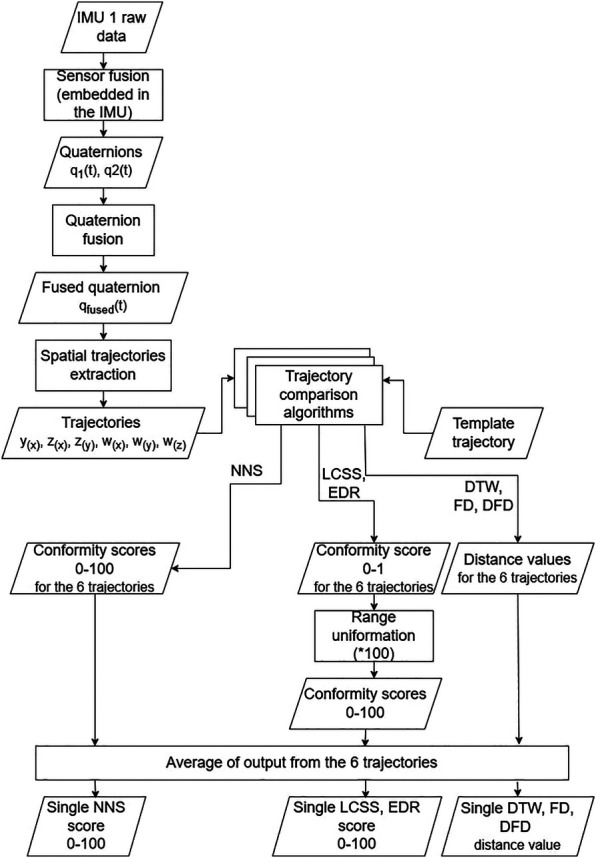
Flowchart of the data processing performed for each acquired trajectory.

Hereafter we describe in detail the data processing performed to obtain outcomes from the algorithms and compare performances. An overview of the process is provided in Figure 3.

The data were processed using Python 3.11, with Numpy 1.26 and Pandas 2.1 for calculations and Matplotlib 3.8 for data plotting.

The data processing was aimed at comparing each of the trajectories acquired in data collection process with a selected *template* trajectory.

For the on-robot test, the first acquisition at the *normal* speed for each movement was used as the *template* to which every of the ten repetitions for each speed was compared, obtaining a distribution of ten outputs for each algorithm at every speed.

For the on-person test, for each individual, a randomly selected repetition of each movement was used as the *template* for the considered movement. The other nine repetitions for every movement were compared to the templates of the movement itself and of all the other movements. For each comparison, the outcomes from the nine repetitions were averaged. Thus, a matrix of comparisons was obtained, with auto-comparisons on the diagonal and cross-comparisons outside the diagonal.

The extraction of the comparison outcomes was performed as in Figure 3 and is detailed hereafter.

Starting from the two quaternions $q_{1}$ and $q_{2}$ provided by each of the two IMUs, the *fused* quaternion $q_{\mathrm{fused}}$ was calculated as in Equation 3 to obtain the relative orientation between the sensors:

(3) $$q_{\mathrm{fused}}=q_{1}q_{2}^{*}$$

All analyses were performed using $q_{\mathrm{fused}}$. For both the on-robot and on-person tests, the NNS and algorithms considered in (49) were applied to the six quaternion component trajectories listed in Equation 2. The algorithms in (49) were imported into Python relying on the repository by Guillouet and Van Hinsbergh (66). NNS was implemented using Python 3.11.5. For the algorithms with bounded output considered in (49) (EDR, LCSS), the threshold for considering two points as similar was set to 0.025, which corresponds to an equivalent variation of $2.86^{\circ }$ in Euler angles, which is approximately the error tolerance of our measurement instrument (34). For the same reason, in the NNS the threshold $t_{\mathrm{acc}}$ was set to the same value 0.025. Outputs from the EDR and LCSS typically range on opposite scales (that is, EDR outputs 0 for identical trajectories, and 1 for completely different trajectories, and LCSS viceversa). However, the Python implementation that we used (66) aligns the output of the two algorithms, so that both range between 0 and 1, with 1 being the output for identical trajectories for both algorithms. In addition, we multiplied the output from these two algorithms by 100 to obtain a conformity score comparable to the results from the NNS. Thus, the outputs from all three bounded algorithms ranged between 0 and 100, with 100 being the output for two identical trajectories.

The metrics obtained for each of the six quaternion components trajectories were then averaged to obtain a single conformity indicator for each algorithm.

After concluding the process of extracting a comparison outcome for each acquired trajectory, we proceeded with the statistical analysis of the results. Descriptive statistics was performed in Python 3.11, while statistical tests were run in R 4.4.3.

For the on-robot test, we constructed the distributions of the ten outcomes from each algorithm at each considered speed.

For the on-person test, based on the obtained comparison matrices, distributions for both values along the diagonals and values outside of it were constructed for each of the considered algorithms. Normality was evaluated on each distribution with a Shapiro-Wilk test.

For normal distributions, a two-sided t-test was performed, whereas for non-normal distributions a two-sided Mann-Whitney U-test was applied, to test the hypothesis of dissimilarity between diagonal and off-diagonal distributions. The Cohen’s *d* or the absolute value of rank-biserial correlation (*rrb*) were used as effect sizes for the t-test or Mann-Whitney U-test, respectively.

Next, the average comparison matrix across all participants was calculated.

To verify the contrast of the average comparison matrix, the values in the average comparison matrix were normalized to the maximum value in the matrix. Because the unbounded algorithms output higher values when the dissimilarity between movements increases, and vice versa for the bounded algorithms, we aligned these two behaviors by subtracting the normalized matrices of the unbounded algorithms from 1. Considering the normalized matrices, the average of values on the diagonal and the average of values outside the diagonal were then calculated. The ratio between the average on the diagonal and the average outside the diagonal was then calculated as Equation 4:

(4) $$r=\frac{mean\;(diag)}{mean\;(outsidediag)}$$

The *r* ratio was calculated separately for each of the two age groups in the testing population, and finally an overall average ratio was calculated.

An analysis of the performance of the NNS algorithm compared to the others was also performed. In order to be comparable, values in the comparison matrices for each participant and each algorithm were rescaled in a 0–100 scale, with 0 representing the minimum value and 100 the maximum, based on Equation 5:

(5) $${value}_{scal}=\frac{100}{max(M)-min(M)}*({value}_{orig}-min(M))$$

where M represents the overall matrix, value_scal_ the rescaled value, and value_orig_ the original value.

After rescaling, the distributions of values on diagonals and outside diagonals were constructed. Normality of the distributions was evaluated through Shapiro-Wilk tests. Next, the distribution on the diagonal from the NNS algorithm was compared with the distribution on the diagonal from each of the other algorithms through a two-sided t-test in case of normality, or a Mann-Whitney U-test in case of non normality. The Cohen’s *d* or the rank-biserial correlation (*rrb*) were used as effect sizes for the t-test or Mann-Whitney U-test, respectively. The same process was repeated also for distributions of values outside the diagonals.

## 4 Results

### 4.1 On-robot test

**Figure 4:**
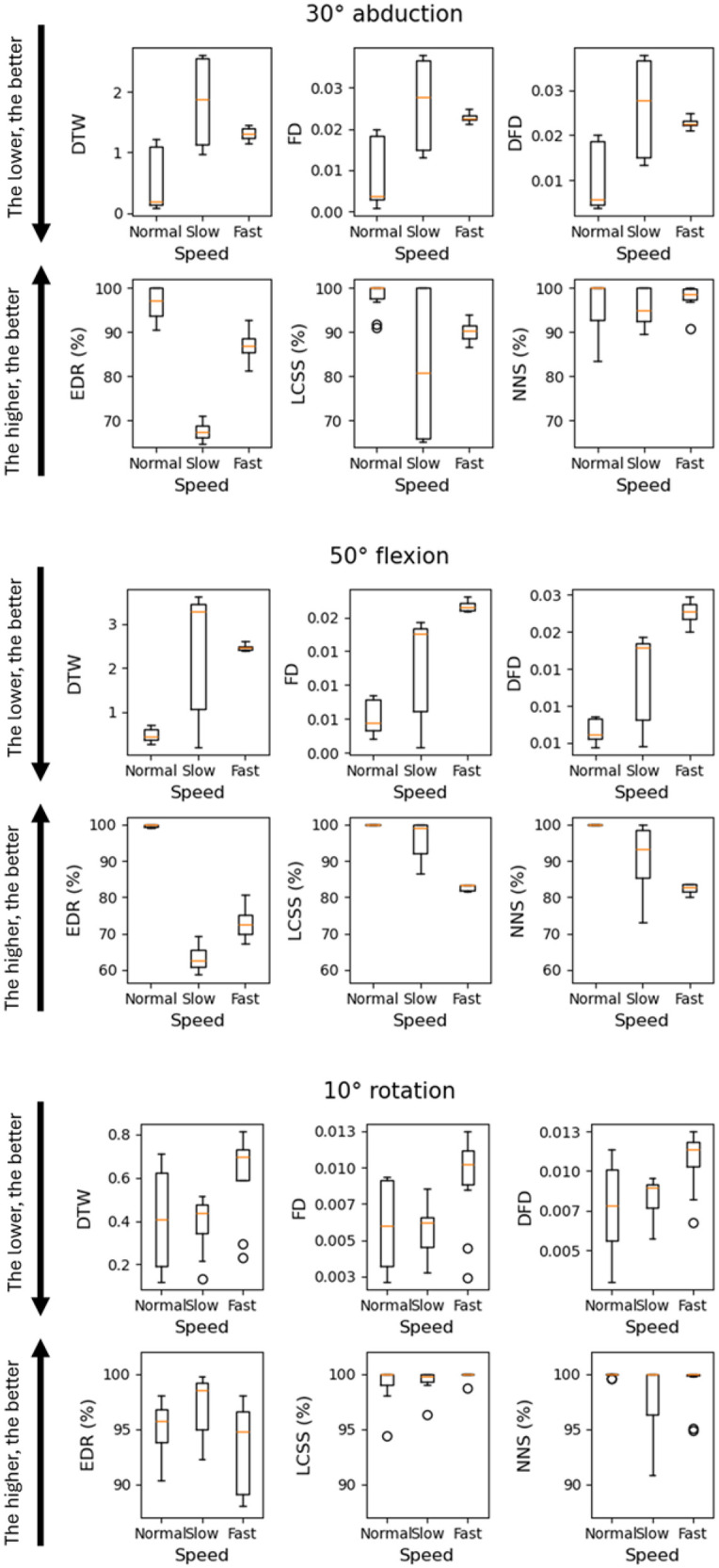
Boxplot representation of distributions of the outputs from the various algorithms obtained when comparing the same planar robotic movements performed at different speeds.

Figure 4 shows the distributions of the outputs from the various algorithms obtained when comparing the same planar robotic movements performed at different speeds.

### 4.2 On-person test

#### 4.2.1 Number of participants

We included 25 participants for the first age group (average age ± standard deviation: 26.1±2.9 years) and 11 participants for the second age group (average age ± standard deviation: 68.1±5.2 years).

#### 4.2.2 Algorithms outcomes

**Figure 5:**
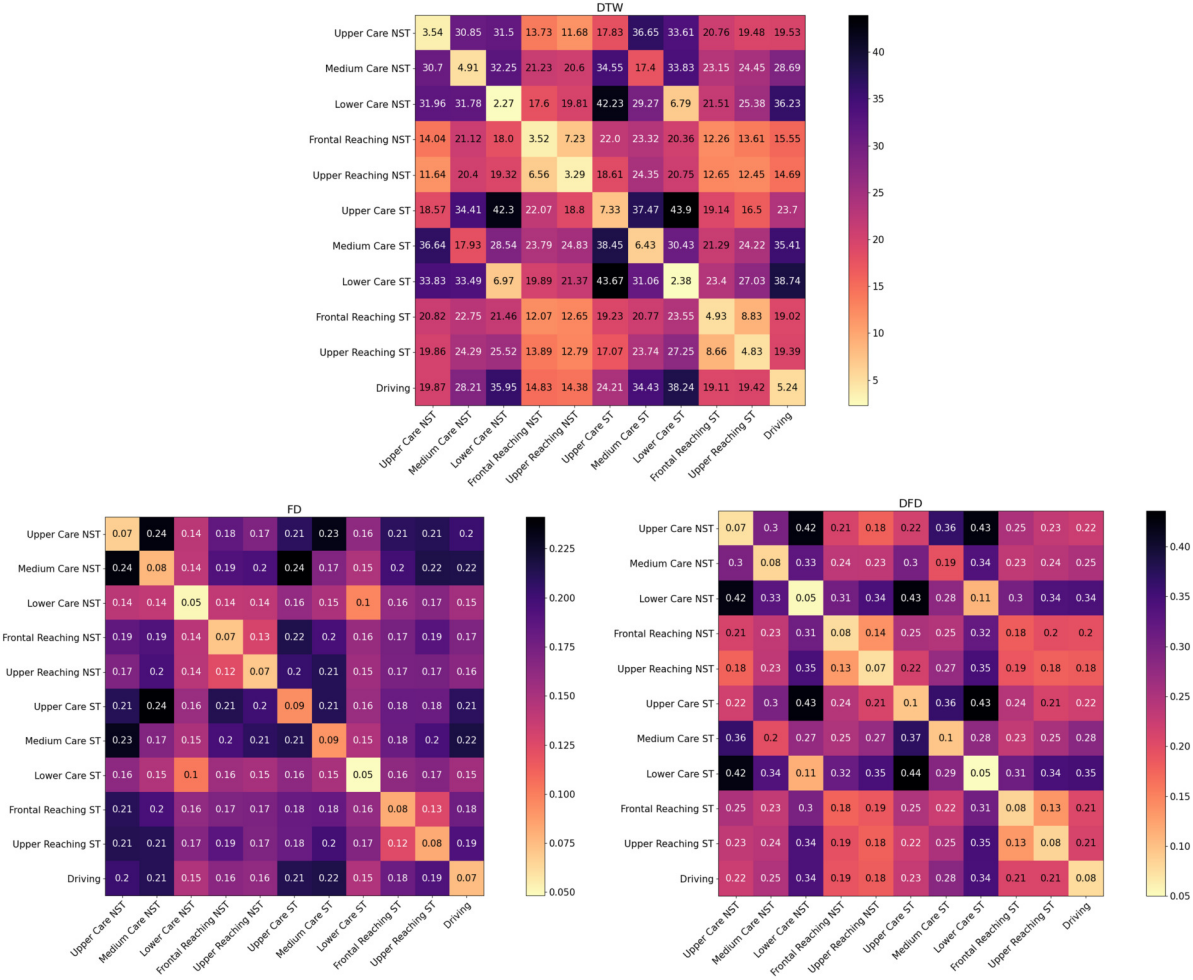
Heatmaps for the average comparison matrices for the unbounded algorithms, obtained by comparing the first repetition of each movement with the other nine, for both non-standardized (NST) and standardized (ST) movements. Values in the cells are average accross compared repetitions and participiants.

**Figure 6:**
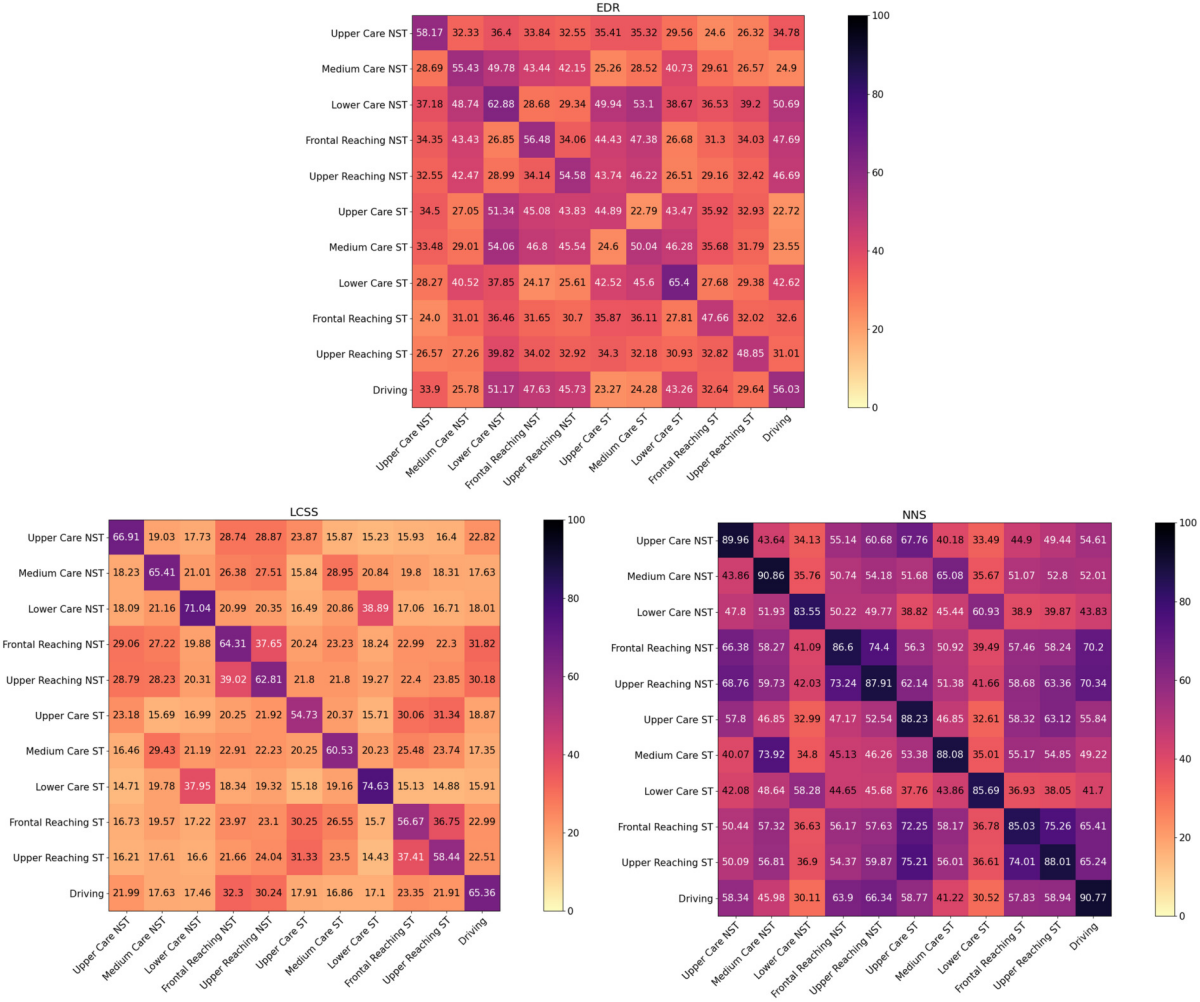
Heatmaps for the average comparison matrices for the bounded algorithms, obtained by comparing the first repetition of each movement with the other nine, for both non-standardized (NST) and standardized (ST) movements. Values in the cells are average accross compared repetitions and participiants.

Figures 5, 6 show heatmaps for the average comparison matrices across subjects obtained when applying the various algorithms to cross-compare the movements performed during the on-person tests. The diagonal in the matrices represents auto-comparison, i.e., average outputs from the comparison of repetitions of a movement with a template of the same movement. Values outside the diagonal represent cross-comparisons, i.e., average outputs from the comparison of repetitions of a movement with a template of a different movement.

#### 4.2.3 Performance indicators

Table 2 shows the *r* ratios, calculated as in Equation 4, obtained for each of the considered algorithms for both the young and elderly groups, and for the overall population. *r* ratios are all >1.

**Table 2:** Ratios *r* between average on diagonal and outside diagonal of obtained comparison matrices for the considered algorithms for both the young and elderly population, and overall.

Parameter	DTW	FD	DFD	EDR	LCSS	NNS
*r* (overall)	1.91	2.67	2.11	1.55	2.88	1.70
*r* (young group)	2.01	2.32	2.16	1.41	2.78	1.66
*r* (elderly group)	1.81	3.0	2.02	1.69	2.99	1.74

All Shapiro-Wilk tests applied to distributions obtained from values on the diagonals and outside the diagonals of the comparison matrices of all subjects exhibited p<0.05; therefore, all distributions were assumed to not be normal, and Mann-Whitney tests were used to test the hypothesis of dissimilarities between the distributions.

Table 3 shows *p* values, effect size (absolute value of rank-biserial correlation, *rrb*), 95% confidence interval (CI) and test power for the Mann-Whitney U test comparing distributions of values on the diagonals and outside the diagonals. Mann-Whitney tests exhibited p<0.05 for every algorithm: therefore, the null hypothesis was rejected for every algorithm. *rrb* effect sizes are all >0.9 (with the exception of EDR being however still >0.5), indicating a strong difference between the distributions. Therefore, we can assume that distributions of values outside the diagonal significantly differ from the distribution of values on the diagonal for every algorithm.

**Table 3:** *p* values, effect size (absolute value of rank-biserial correlation, *rrb*), 95% confidence interval (CI) and test power obtained from Mann-Whitney test comparing distributions on diagonal and outside diagonal.

Algorithm	*p* value	Effect size (*rrb*)	95% CI	Test power
DTW	$<0.01^{*}$	0.96	[0.95,0.96]	1
FD	$<0.01^{*}$	0.95	[0.94,0.95]	1
DFD	$<0.01^{*}$	0.98	[0.97,0.98]	1
EDR	$<0.01^{*}$	0.73	[0.71,0.76]	1
LCSS	$<0.01^{*}$	0.97	[0.97,0.98]	1
NNS	$<0.01^{*}$	0.97	[0.97,0.97]	1

Asterisks indicate p<0.05.

All Shapiro-Wilk tests applied to distributions rescaled in 0–100 range from values on the diagonals and outside the diagonals of the comparison matrices of all subjects exhibited p<0.05; therefore, all distributions were assumed to not be normal, and Mann-Whitney tests were used to compare distributions from the NNS algorithm with the ones from the other algorithms.

Table 4 reports *p* values, effect size (rank-biserial correlation, *rrb*), 95% confidence interval (CI) and test power for the Mann-Whitney U test comparing the rescaled distributions of NNS on the diagonal with the rescaled distributions on diagonals from the other algorithms. Table 5 reports results from the same tests applied on rescaled distributions of values outside the diagonal. All tests exhibited statistically significant difference (p<0.05) both on diagonal and outside diagonal, with the only exception of DFD on diagonal, where no statistically significant difference was found. However, effect sizes were very contained for DTW and FD, indicating a mild difference. On the contrary, significant effect sizes were obtained for comparison EDR and LCSS, with *rrb* assuming negative values, indicating higher ranks associated with NNS.

**Table 4:** *p* values, effect size (rank-biserial correlation, *rrb*), 95% confidence interval (CI) and test power obtained from Mann-Whitney test comparing scaled distributions on diagonal of NNS with other algorithms.

Algorithm	*p* value	Effect size (*rrb*)	95% CI	Test power
DTW	$<0.01^{*}$	0.21	[0.14, 0.28]	0.87
FD	$<0.01^{*}$	−0.34	[−0.41, −0.27]	1
DFD	0.21	−0.05	[−0.13, 0.03]	0.11
EDR	$<0.01^{*}$	−0.69	[−0.73, −0.65]	1
LCSS	$<0.01^{*}$	−0.55	[−0.60, −0.49]	1

Asterisks indicate p<0.05. Negative values of *rrb* indicate higher ranks associated with NNS.

**Table 5:** *p* values, effect size (rank-biserial correlation, *rrb*), 95% confidence interval (CI) and test power obtained from Mann-Whitney test comparing scaled distributions outside diagonal of NNS with other algorithms.

Algorithm	*p* value	Effect size (*rrb*)	95% CI	Test power
DTW	$<0.01^{*}$	0.36	[0.34, 0.38]	1
FD	$0.025^{*}$	−0.03	[−0.05, 0]	0.03
DFD	$<0.01^{*}$	0.13	[0.11, 0.16]	1
EDR	$<0.01^{*}$	−0.11	[−0.13, −0.08]	1
LCSS	$<0.01^{*}$	−0.97	[−0.98, −0.97]	1

Asterisks indicate p<0.05. Negative values of *rrb* indicate higher ranks associated with NNS.

## 5 Discussion

### 5.1 Algorithm performance and applicability

The numerical stability of the analysis in quaternion space is confirmed by the traditional unbounded algorithms’ capability of detecting similarities and differences between movements, as supported by the good *r* ratios and low *p* values with high effect sizes obtained when assessing contrast of DTW, FD and DFD.

However, bounded algorithms can provide several advantages from the point of view of user experience and patient engagement, as they can directly provide a conformity score without the need for any additional information about the movement being performed (e.g., for estimating boundary conditions to constrain the output).

To the best of our knowledge, this is the first study to explore the application of bounded algorithms in shoulder functional rehabilitation. Our results suggest the applicability of bounded algorithms to the assessment of shoulder rehabilitation exercises, combined with an analysis in the quaternion space for numerical stability.

All bounded algorithms, in fact, performed acceptably in detecting similarities and differences between movements, with *r* ratios >1.5, low *p* values and high effect sizes (comparable to the ones from unbounded algorithms) in statistical tests for contrast performance assessment. It is, however, worth discussing some specificity for each one that is supported by the results we obtained, especially how our new NSS proposal collocates in this context.

Among these algorithms, EDR demonstrated to be the worst performer, with the lowest contrast ratio and smaller effect size in statistical tests. This behavior, apparently worse than LCSS, could be explained by a different impact of the acceptance threshold on the final scoring because of a different main goal of the algorithm. Whereas EDR extracts the final score by thresholding a cost matrix similar to that of DTW, the LCSS implementation is conversely aimed at finding the maximum score path (49).

It is interesting to analyze the performance of our new NNS proposal in comparison to the other algorithms. Looking at the *r* contrast ratio, NNS performs better than EDR and comparable to DTW, while FD, DFD and LCSS exhibit better contrast. It is however worth noting that NNS is the algorithm with lowest difference in contrast ratio between the two age groups, suggesting a better capability to generalize the evaluation among a variety of motor strategies. This supports the potential of comparing the trajectories analytically through a distance-based scoring method: in fact, fluctuations in contrast are possible in DTW, FD and DFD algorithms due to their unbounded nature, which allows for the output to increase unlimitedly. This consideration is further supported by statistical tests on rescaled distributions: when rescaled in same range based on empirical maximum and minimum in the data, the comparison between NNS and unbounded algorithms (DTW, FD, DFD) leads to small *rrb* effect sizes, suggesting equal performance due to comparable output.

Among bounded algorithms, both the *r* ratio and the statistical tests indicate improved performance with respect to EDR. In comparison to LCSS, *r* ratio of NNS is lower, while LCSS contrast even outperforms the unbounded algorithms, indicating a very good resolution in the discrimination between the same and different movements. However, in the LCSS average matrix, the maximum value obtained was 63.02, which is quite far from the maximum possible output of the algorithm (100). On the contrary, looking at results from robotic data, it is visible that in controlled conditions the output when comparing the same movements is close to 100 in most cases. The statistical tests comparing rescaled distributions of NNS with the ones of LCSS indeed led to significant results, with low *p* value and a high *rrb* effect size with negative values, indicating higher ranks for the NNS both on the diagonal and outside diagonal.

This suggests that, when used on human movement data, LCSS, despite being able to detect with good contrast the dissimilarities between movements, it always detects some differences, even when comparing repetitions of the same movements. In other words, the algorithm classifies repetitions of the same human movement as *more similar* to each other than when comparing different human movements, but not as *almost identical*.

Conversely, our new NNS proposal, detects repetitions of the same movements as *almost identical*, while detecting comparisons of different movements as *less similar* than the auto-compared ones, still with an acceptable *r* contrast ratio and performance that was statistically demonstrated to be comparable to the ones of the unbounded algorithms.

The choice of one or the other algorithm might therefore be based on the intention of focusing mostly on penalizing incorrect movements, or mostly on awarding well-performed movements, depending on the clinical condition of the patient and on the type of treatment.

### 5.2 Potential impact of the results

Most studies available in the literature dealing with telerehabilitation systems exploit unbounded algorithms, mainly DTW, to assess the exercises and provide feedback to the user. Nonetheless, to make this feedback intuitive for the user, some form of transformation of the unbounded output is necessary, typically a conversion to a conformity score. This transformation often involves the intervention of experimental estimation or suggestions from clinicians to define boundaries, or the discretization of the output, thereby losing the potential versatility of the template-based approach.

As examples, (67) elaborated the DTW output using a sigmoid function tuned relying on indications from clinicians and defining a priori rules; (43) used an upper limit output estimated from experimental data to transform DTW output into a score; (61) converted DTW output in discrete levels (e.g., “Bad,” “Good,” “Excellent”) using fuzzy logic; (57) converted DTW output into a conformity score, but still needed calibration of the algorithm using ratings from experts on a subset of participants. Among the most recent works, Beaud et al. (58) developed a DTW-based score, which, however, has a lower bounded constraint (the closer the score is to 0, the most similar are the trajectories), but still no constraint on upper boundary. Seo et al. (62) used DTW to evaluate quality of upper limb tasks, but falled into a simple binary categorization into “Correct” or “Incorrect.” Pereira et al. (59) transformed DTW output into a z-score, which, however, required information (mean and standard deviation) about their specific dataset.

Therefore, in all these cases, some form of experimental inference about the movement or of output discretization is performed, but this limits the potential of tele-rehabilitation as a tool that fosters the independence of the patient and allows for personalized treatment.

Also, recent studies focus on the use of data-driven probabilistic approaches, given the high potential of recent advancements in machine and deep learning (31, 42, 43, 46, 68). Those approaches are very powerful when it is necessary to recognize or classify specific movements (68): however, they require a large amount of data to be trained, and need to be retrained if it is necessary to change the template trajectory, while the advantage of distance-based methods is that they are not exercise-specific (68). In fact, in functional rehabilitation, it is essential to define the movements that need to be performed based on the specific condition of the patient, and those might vary depending on the specific pathology, the clinical progress and even the psico-social context in which the patient is immersed (10–13, 22–24). Therefore, it is important to ensure that new template trajectories can be defined at any moment during the progress of the treatment for the specific patient, without needing to retrain the comparison algorithms.

The calculation of a conformity score through trajectory comparison algorithms with a bounded output, such as the NNS proposed in this study, allows for more personalized treatment. These algorithms only take as input a template trajectory (whichever being its shape) and a repetition of the exercise, and directly output a conformity score in a limited range (e.g., 0–100) quantifying the similarity between the two movements.

This is very important to introduce in telerehabilitation the potential for a patient-centric personalized treatment. In this way the clinician can define a template trajectory based on the clinical condition of the patient. Next, the assessment algorithm will compare the at-home performed repetitions of the movement, without the need for any experimental estimation of the boundaries, any previous training dataset collection, or any form of discretization of the output.

Quaternion-based assessment adds numerical stability, which allows to avoid calibration procedures that are often performed when working with angle-based approaches to estimate values in known positions (e.g., T-pose) (69).

Moreover, bounded algorithms rely on a distance threshold for the classification of points in a trajectory as either similar or different to those in the template trajectory. This threshold can become an important tool in telerehabilitation. Threshold tuning can be used by the clinician to set the amount of accepted error in movement execution, based on the specific condition of the patient. This is another advantage in the direction of a patient-centric remote rehabilitation treatment.

### 5.3 Limitations and future directions

This study explored the application of bounded algorithms for trajectory comparison in the quaternion space during the execution of shoulder functional rehabilitation exercises, introducing a new algorithm for the comparison and confronting its performance with existing ones.

The results demonstrated the applicability of the quaternion-based approach and the reliability of using bounded algorithms, including our new proposal, considering the potential flexibility that they offer compared to the unbounded ones.

However, this study has some limitations. First, the sample size could be further enlarged to strengthen the relevance of the results obtained. Specifically, we tested on a group of young healthy participants and a limited group of elderly participants, but including actually impaired participants may provide further insights on how the methodology responds in persons with actual changes in kinematic trajectories. It is expected that the output of the algorithms would be akin to when comparing similar but slightly different movements (e.g., Frontal and Upper Reaching), due to the performance of a similar movement but with compensatory behavior. However, this might be pathology-dependent: for example, a patient with an orthopedic impairment that causes pain in a very specific area might present a very specific compensatory phase, while patients with neurological impairment might present a constant tremor or a different trajectory planning, and the various considered algorithms could respond differently in these two situations.

Moreover, because the goal was to verify the applicability of such a method as an assessment tool based on data from wearable devices, we directly tested on IMU data: nonetheless, because no comparison were made with data collected simultaneously from gold standard systems (e.g., the Vicon), the actual similarity accross the various repetitions of movements could be ulteriorly strengthened. We previously ensured the functionality of the method on identical movements through the tests on the robotic arm, but further developments of this work might add analysis with gold standard instruments to reinforce the assessment of identical movements. In fact, while IMU were proven to be reliable sensors for upper limb motion analysis (15, 16, 34), it was also demonstrated that their error is larger when compared to optoelectronic systems (70, 71): therefore, we aknowledged this limitation by previously testing on robotic arm and by inserting the measurement accuracy threshold in the NNS algorithm, but a future comparison of trajectories simultaneously acquired also through gold standard systems might ensure the actual conformity between movements.

In addition, implementing the algorithm in a basic system that can be used by a sample of elderly or impaired users might provide more insights into the actual usability of the approach. This is a future direction that should be pursued to further improve the applicability of the approach in real scenarios.

We also defined only a subset of possible functional movements involving the shoulder joint; however, future directions may involve widening the number and type of movements to strengthen the applicability of the proposed approach.

Other insights could arise from an analysis of the influence of the choice of threshold for bounded algorithms on the resulting conformity scores. This may further clarify the impact that the implementation of bounded algorithms can have on telerehabilitation systems, and also provide insights about the feasibility of using the threshold as a tool for the clinician to tune the accepted error in movement performance based on the patient condition.

## 6 Conclusion

This study explored the feasibility of comparing repetitions of upper limb functional movements to a template by analyzing trajectories in the quaternion space. A novel algorithm for trajectory comparison that produces a bounded conformity score was applied.

This approach effectively identified both coherence and dissimilarities in shoulder functional exercise repetitions, demonstrating the reliability of quaternion-based analysis and bounded algorithms for trajectory comparison. This new approach has some practical advantages, including improved personalization of remote rehabilitation treatments.

Future work could involve implementing the proposed approach in a real system to obtain feedback from end-users in practical application scenarios.

## Data Availability

The raw data supporting the conclusions of this article will be made available by the authors, without undue reservation. The code of the proposed NNS algorithm and a subset of the data used for experimental validation can be found in the NNS algorithm repository https://github.com/iurmat/NNS_algorithm.git.
