# Supplementary material for: Assessment of shoulder functional movements through inertial measurement units for tele-rehabilitation: a quaternion-based approach
Source: Front Digit Health. 2025 Sep 1;7:1576031. doi: 10.3389/fdgth.2025.1576031 (PMC12434763; doi:10.3389/fdgth.2025.1576031)
Supplement: Supplementary file 1 [file Datasheet1.pdf]

Supplementary Material

1 TRAJECTORIES USED FOR THE ANALYSIS

Figures 1, 2, 3 show the robotic trajectories used for analysis at various speed. Figure 4 shows an example of human trajectories (for non-standardized Upper Care movement).

REFERENCES

Stanzani R, Dondero P, Mantero A, Testa M. Measurement accuracy of an upper limb tracking system based on two hillcrest labs bno080 imu sensors: An environmental assessment. *IEEE Sensors Journal* **20** (2020) 10267–10274.

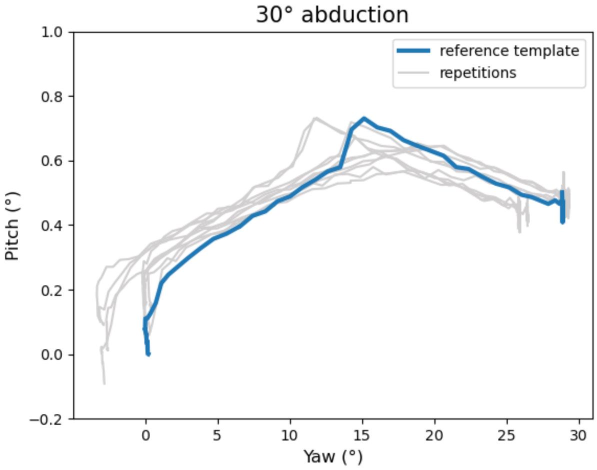

Figure 1a. Trajectory in Yaw-Pitch plane. The initial offset of the *template* curve was removed from all trajectories.

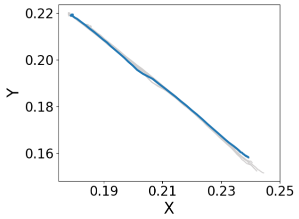

Figure 1b. $y_{(x)}$ quaternion trajectory

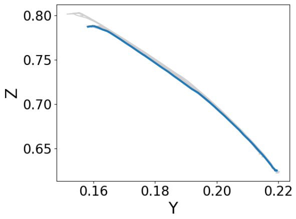

Figure 1c. $z_{(y)}$ quaternion trajectory

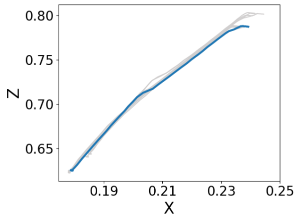

Figure 1d. $z_{(x)}$ quaternion trajectory

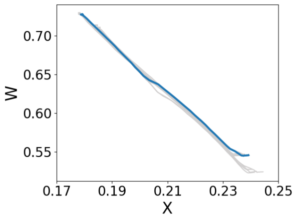

Figure 1e. $w_{(x)}$ quaternion trajectory

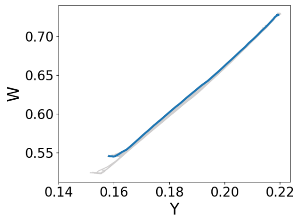

Figure 1f. $w_{(y)}$ quaternion trajectory

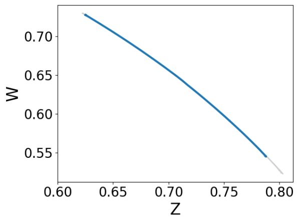

Figure 1g. $w_{(z)}$ quaternion trajectory

Figure 1. Trajectories of the $30°$ abduction robotic movement.

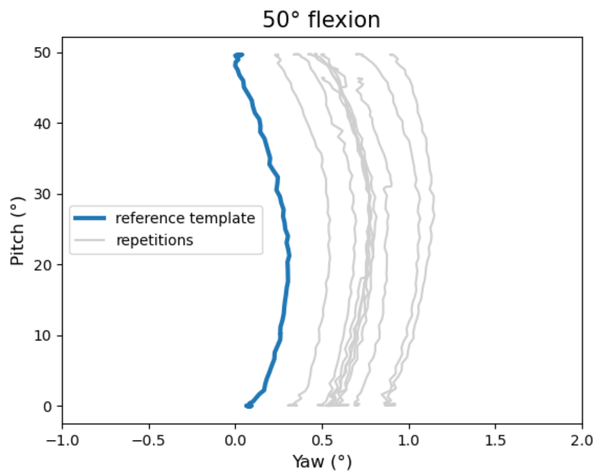

Figure 2a. Trajectory in Yaw-Pitch plane. The initial offset of the *template* curve was removed from all trajectories. A drift of $< 1°$ is visible in the Yaw angle, which is consistent with the findings in Stanzani et al. (2020).

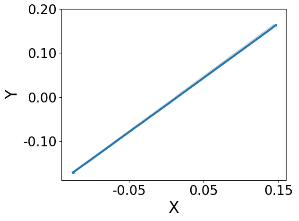

Figure 2b. $y_{(x)}$ quaternion trajectory

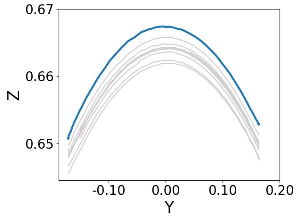

Figure 2c. $z_{(y)}$ quaternion trajectory

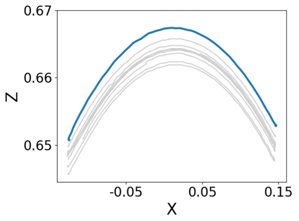

Figure 2d. $z_{(x)}$ quaternion trajectory

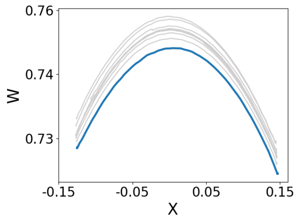

Figure 2e. $w_{(x)}$ quaternion trajectory

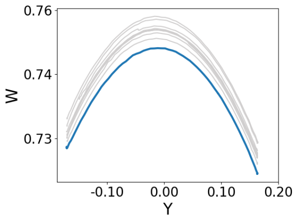

Figure 2f. $w_{(y)}$ quaternion trajectory

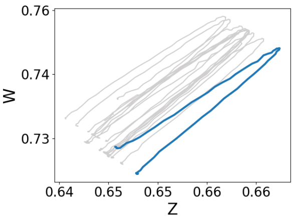

Figure 2g. $w_{(z)}$ quaternion trajectory

Figure 2. Trajectories of the $50°$ flexion robotic movement.

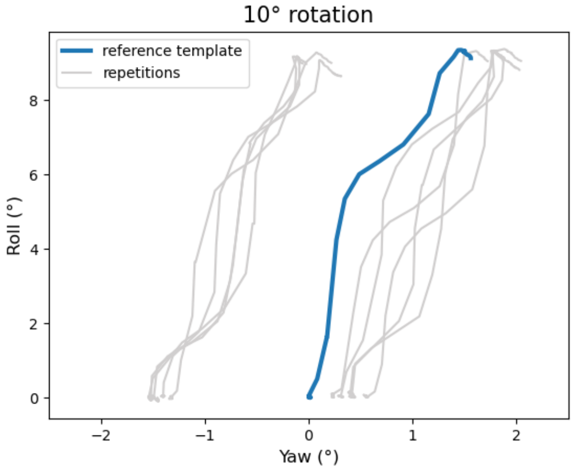

Figure 3a. Trajectory in Yaw-Roll plane. The initial offset of the *template* curve was removed from all trajectories. A drift of $< 2°$ is visible in the Yaw angle, which is consistent with the findings in Stanzani et al. (2020).

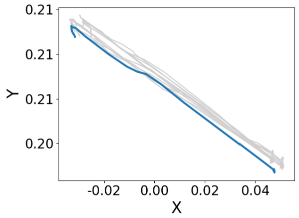

Figure 3b. $y_{(x)}$ quaternion trajectory

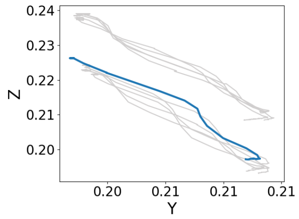

Figure 3c. $z_{(y)}$ quaternion trajectory

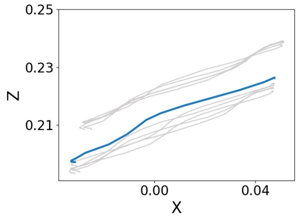

Figure 3d. $z_{(x)}$ quaternion trajectory

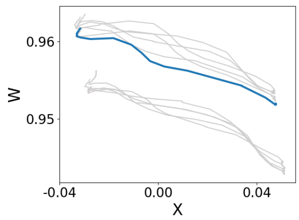

Figure 3e. $w_{(x)}$ quaternion trajectory

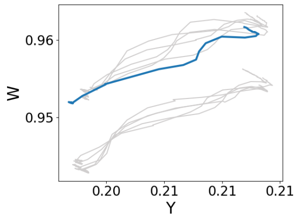

Figure 3f. $w_{(y)}$ quaternion trajectory

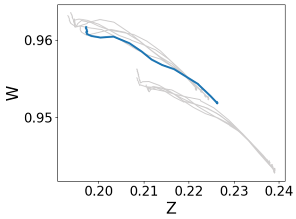

Figure 3g. $w_{(z)}$ quaternion trajectory

Figure 3. Trajectories of the $10°$ rotation robotic movement.

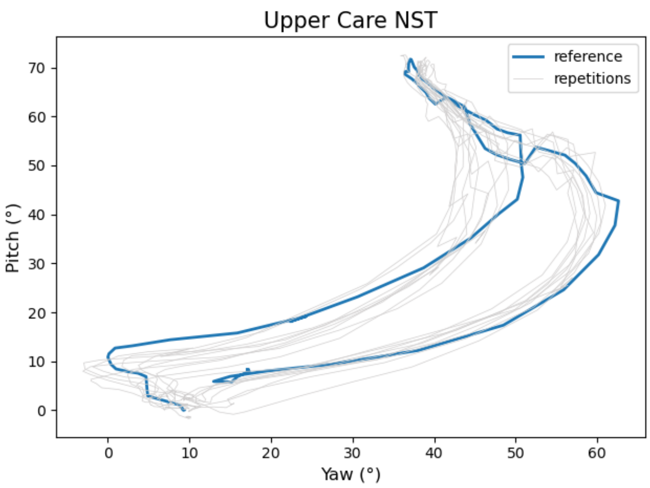

Figure 4a. Trajectory in Yaw-Pitch plane. The initial offset of the *template* curve was removed from all trajectories.

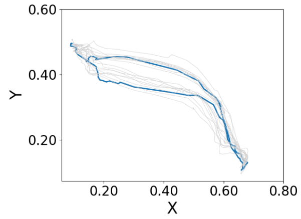

Figure 4b. $y_{(x)}$ quaternion trajectory

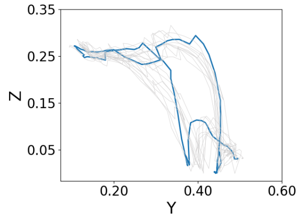

Figure 4c. $z_{(y)}$ quaternion trajectory

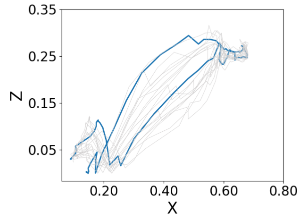

Figure 4d. $z_{(x)}$ quaternion trajectory

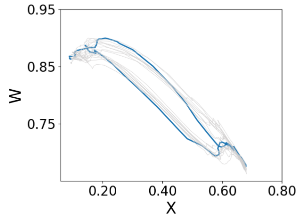

Figure 4e. $w_{(x)}$ quaternion trajectory

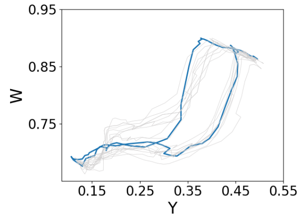

Figure 4f. $w_{(y)}$ quaternion trajectory

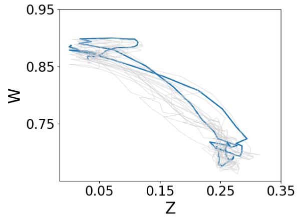

Figure 4g. $w_{(z)}$ quaternion trajectory

Figure 4. Trajectories of Upper Care non-standardized (NST) human movement.
